# Effects of Printing Parameters on the Fit of Implant-Supported 3D Printing Resin Prosthetics

**DOI:** 10.3390/ma12162533

**Published:** 2019-08-09

**Authors:** Gang-Seok Park, Seong-Kyun Kim, Seong-Joo Heo, Jai-Young Koak, Deog-Gyu Seo

**Affiliations:** 1 Department of Prosthodontics and Dental Research Institute, Seoul National University Dental Hospital, School of Dentistry, Seoul National University, Seoul 03080, Korea; 2 Department of Conservative Dentistry, School of Dentistry, Seoul National University, Seoul 03080, Korea

**Keywords:** implant, prosthesis, 3D printing, fit, bioengineering

## Abstract

The purpose of the study was to investigate the influence of 3D printing parameters on fit and internal gap of 3D printed resin dental prosthesis. The dental model was simulated and fabricated for three-unit prostheses with two implants. One hundred prostheses were 3D printed with two-layer thicknesses for five build orientations using a resin (NextDent C&B; 3D systems, Soesterberg, The Netherlands) and ten prostheses were manufactured with a milling resin as control. The prostheses were seated and scanned with micro-CT (computerized tomography). Internal gap volume (IGV) was calculated from 3D reconstructed micro-CT data. IGV, marginal fit, and lengths of internal gaps were measured, and the values were analyzed statistically. For the 3D printed prostheses, IGV was smaller at 45°, 60°, and 90° compared to other build orientations. The marginal fit evaluated by absolute marginal discrepancy was smaller than other build orientations at 45° and 60°. IGV was smaller at 50 µm layer thickness than at 100 µm layer thickness, but the marginal fit was smaller at 100 µm layer thickness than at 50 µm layer thickness. The 3D printed prosthesis had smaller internal gap than the milled prosthesis. The marginal fit of the 3D printed resin prosthesis was clinically acceptable, and build orientation of 45° and 60° would be recommended when considering fit and internal gap.

## 1. Introduction

As computer-aided design/computer-aided manufacturing (CAD/CAM) technology has developed, the CAD/CAM prosthesis has been frequently used in dental clinics [1]. Previously, most of the prosthesis was made using the lost-wax technique, which took a long time to produce the prosthesis, and there was a lot of errors caused by the technician during the manufacturing process [2,3]. When the prosthesis is manufactured by the CAD/CAM method, as it takes less time than the conventional method and the prosthesis can be manufactured more reproducibly [4].

There are additive manufacturing and subtractive manufacturing processes in CAM. Additive manufacturing, also called three-dimensional (3D) printing, is a method of stacking materials one by one. Subtractive manufacturing, also known as milling, is a method of cutting chunks of material into a desired shape. Although there are advantages and disadvantages to either method, additive manufacturing has many advantages such as less waste of material and less generation of heat and noise when manufactured compared to the subtractive method. In addition, there is no need for the bur to be worn and replaced, or unnecessary force being applied to the material, which occurs in the milling [5,6,7].

Among the various techniques for additive manufacturing, digital light processing (DLP) is increasingly used for dental prosthesis manufacturing [8]. It uses laser to polymerize the liquid and digital mirror device (DMD) controls the curing laser. DMD is made up of a number of micro-mirrors, which control the operation of the laser beam as they move independently. DLP is faster than the conventional stereolithography (SLA) process because it can cure an entire layer at a time using the digital mirror device [9].

In recent years, interim restorations have been made with CAD/CAM technique [10], and implant provisional restorations have also been produced using DLP method [8]. Interim restorations are used to restore function and esthetics and evaluate outcomes before final restorations [11]. They protect the abutments, give appropriate occlusal scheme [12], and help soft tissue healing by molding and shaping the soft tissue [13]. To achieve these goals, fit of the restorations should be accurate and well-evaluated [11,14]. Inadequate marginal fit can cause cement loss, plaque accumulation, periodontal disease and aesthetic appearance damage [11].

Previous studies have evaluated the marginal fit of 3D printed prosthesis made at a fixed build orientation [10,15]. There were studies comparing the accuracy according to the 3D printing build orientations with the rectangular resin specimen instead of the prosthesis [16,17]. Also, three-dimensional superimposition analysis was performed to compare the dimensional accuracy of the resin crown according to the build orientations [8,18]. However, there has been no study comparing the fit of 3D printing prosthesis according to parameters such as build orientation or layer thickness.

There are various methods to measure the marginal and internal gaps. Direct view technique is the commonly used method of observing the interface between the crown margin and the die using a microscope [19,20]. Also, Cross-sectioning technique [21], impression replica technique [22], optical coherence tomography (OCT) [23], and micro-computed tomography (micro-CT) [24] are used to measure the marginal gap. Among them, OCT uses a low-coherence light and produce high-resolution cross-sectional images [23]. Micro-CT has the merits of repeatedly and precisely measuring at various positions without destroying the specimen [25].

Therefore, the purpose of this study was to investigate the influence of build orientation and layer thickness on marginal fit and internal gap of the resin prosthesis manufactured by 3D printing and to compare them with the fit of prosthesis made by milling. The null hypothesis is that there is no difference in marginal fit and internal gap of prosthesis in different build orientations and layer thickness. Also, there is no difference in marginal and internal fit between the prostheses made by 3D printing and milling.

## 2. Materials and Methods

### 2.1. Model Design and Fabrication

**Figure 1:**
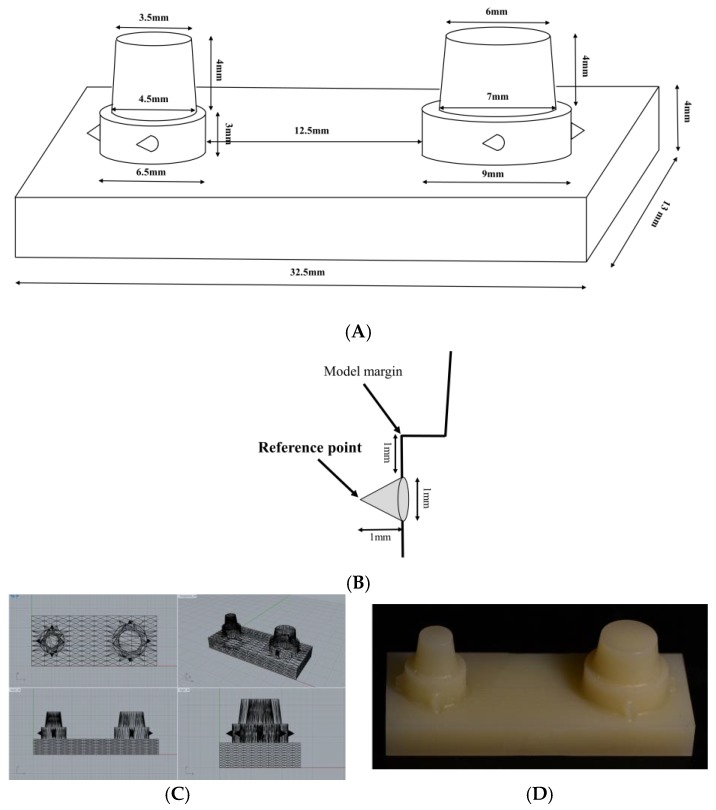
(**A**) The model design for two-implants supported three-unit dental prosthesis, (**B**) design of the reference point in the model, (**C**) computer-aided design (CAD) design of the model in various view and (**D**) the model fabricated with PMMA resin.

A model was designed for two-implants supported three-unit resin dental prosthesis (Figure 1A). Implant customized abutments were located on the mandibular second premolar and second molar area. The first molar area between the two abutments was missing. The specifications of the customized abutments and the spacing between them were determined considering the anatomical size and shape of the teeth [26] and preparations for complete crowns [27]. Abutments were designed with 1 mm shoulder margin. Cone shape reference points with a diameter of 1 mm and a height of 1 mm were designed below the margin of each abutment (Figure 1B). The reference points were extended in three directions with 90° to each other from each abutment.

Designed model converted to standard triangulated language (STL) file using computer-aided design (CAD) software (Rhinoceros 5.0; McNeel, Seattle, WA, USA) (Figure 1C). Using the STL file, polymethylmethacrylate (PMMA) resin block (Yamahachi dental MFG, Ochigara, Japan) was milled with 5-axis milling machine (IDC MILL 5X; Amann girrbach AG, Koblach, Austria) (Figure 1D).

### 2.2. Prosthesis Design and Fabrication

The milled PMMA resin model was scanned with model scanner (Freedom HD; DOF, Seoul, Korea). Scanning accuracy was 10 µm according to manufacturer. Before scanning, the scanner was calibrated following the manufacturer’s instruction, and the model was thinly sprayed with anti-reflective powder (Easy scan; alphadent, Goyang, Korea). Scanned model data were exported in STL file. The three-unit prosthesis was designed using Exocad Dental CAD 2.2 (exocad, Darmstadt, Germany) to fit on scanned model. Cement space was given 0.24 mm except the margin. The cement space of the prosthesis was increased from 0.04 mm to 0.30 mm by 0.02 mm, confirming the fit of the prosthesis on the model. The smallest value that all the specimens were seated passively on the model was 0.24 mm.

**Figure 2:**
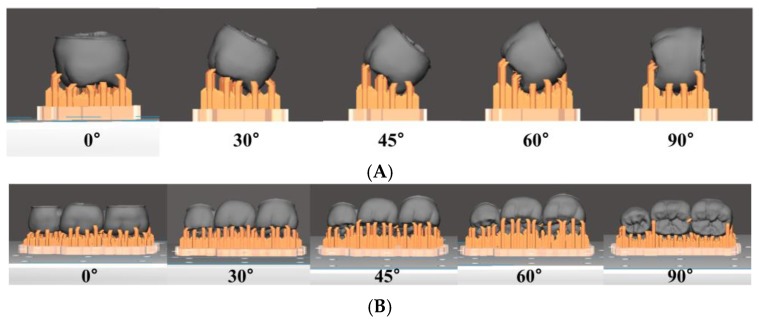
The prosthesis design of five build orientations (0°, 30°, 45°, 60°, and 90°). The supporting structure was connected perpendicular to the occlusal surface of the prosthesis in the 0° build orientation, and was connected perpendicular to the lingual surface of the prosthesis in the 90° build orientation. (**A**) Distal view and (**B**) buccal view of the designed prosthesis.

Designed STL format file used to fabricate 3D printed and milled prosthesis. The prostheses were printed using D2-120 (Hephzibah, Incheon, Korea) DLP printer. The x-y accuracy of the printer was 62.5 µm. The prostheses were printed in five build orientations: 0°, 30°, 45°, 60° and 90° (Figure 2). For each build orientation, prostheses were printed with two-layer thicknesses of 50 µm and 100 µm using NextDent C&B (3D systems, Soesterberg, The Netherlands). Total 10 groups of prostheses were 3D printed with tw- layer thicknesses for five build orientations. 10 prostheses were made for a group and a total of one hundred 3D printing prostheses were made. In the 0° build orientation, supporting structure was connected perpendicular to the occlusal surface of the prosthesis. In the 90° build orientation, supporting structure was connected perpendicular to the lingual surface of the prosthesis. After 3D printing, prostheses were cleaned with 95% ethanol for 2 min, 30 min post-curing was done using ultraviolet curing unit (MP100; Hephzibah, Incheon, Korea) following the manufacturer’s instructions. The milled prostheses were produced using 5-axis milling machine (IDC MILL 5X; Amann girrbach AG, Koblach, Austria). The PMMA resin blocks (Yamahachi dental MFG, Ochigara, Japan) were milled with 0.6-, 1.0- and 2.5-mm diameter burs and ten prostheses were made for milling group. So, a total of one hundred 3D printing prostheses and ten milling prostheses were made. All prostheses were examined under magnification loupe (3.5×) for any defects and no adjustments were made to the inner surface of prostheses. The prostheses were kept in a dry, lightproof box and tested within 5 days after manufacturing.

### 2.3. Micro-CT Scanning

Before seating the prostheses on the model, the model was fixed to the jig for micro-CT scan. This fixation allowed all specimens to be taken at the same position. The prostheses were then seated on the model without cementation. Scanning was performed at 59 kVp and 167 µA, with an exposure time of 1475 ms using desktop micro-CT scanner (Skyscan 1172; Bruker micro-CT, Kontich, Belgium). A 0.5 mm thick aluminum filter was used and the resolution of scan was 15.39 µm. The specimen was rotated 180° with 0.7° rotational step and 3 frames averaging.

### 2.4. Internal Gap Volume Analysis

**Figure 3:**
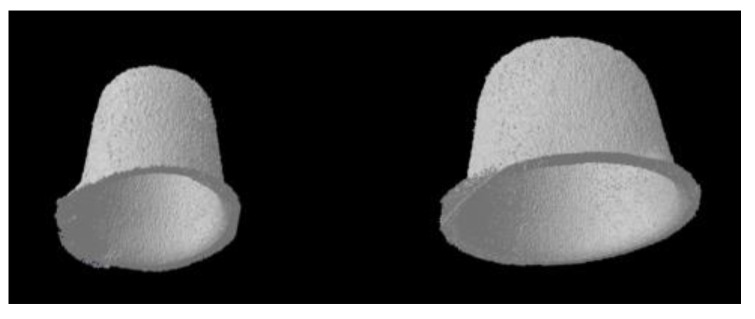
3D reconstructed image of internal gap between the surface of model and the inner surface of prosthesis. The volume of this image was defined as Internal gap volume (IGV).

After micro-CT scanning, data was reconstructed using NRecon v.1.7.3.2 software (Bruker micro-CT, Kontich, Belgium). Smoothening was set to 3, ring artifacts reduction to 8, and beam-hardening correction to 20%. The entire volume between the surface of the model and the inner surface of prosthesis was defined as internal gap volume (IGV). The IGV was calculated using CTAn v.1.17.7.2 (Bruker micro-CT, Kontich, Belgium). 3D reconstructed image of the internal gap between the model and the prosthesis was shown in Figure 3.

### 2.5. Marginal Fit and Internal Gap Length Analysis

**Figure 4:**
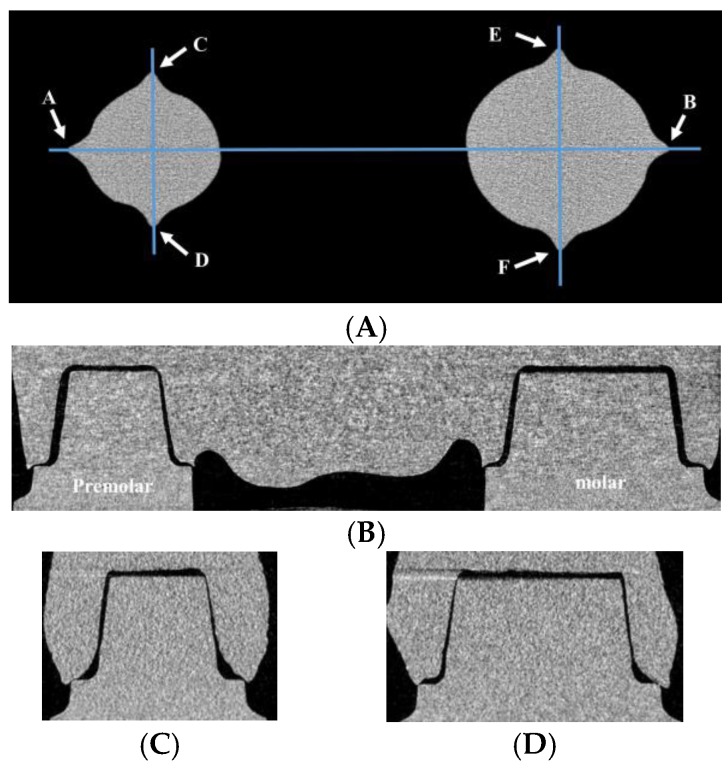
(**A**) A horizontal plane containing reference points A to F on CT data was defined as a reference plane. On the reference plane, a blue line containing reference points A and B indicates the coronal section. Another line containing reference points C and D indicates the premolar sagittal section and the other line containing reference points E and F indicates the molar sagittal section. (**B**) The coronal section, (**C**) the premolar sagittal section and (**D**) the molar sagittal section.

Marginal fits and internal gaps were measured by DataViewer v.1.5.6.2 software (Bruker microCT, Kontich, Belgium) using reconstructed data. A horizontal plane containing reference points A to F was defined as a reference plane (Figure 4A). A section perpendicular to the reference plane including points A and B was defined as a coronal section (Figure 4B). A section perpendicular to the reference plane including points C and D was defined as a premolar sagittal section (Figure 4C). A section perpendicular to the reference plane including points E and F was defined as a molar sagittal section (Figure 4D). Marginal fit and internal gap length were measured in coronal section, premolar sagittal section and molar sagittal section, respectively.

**Figure 5:**
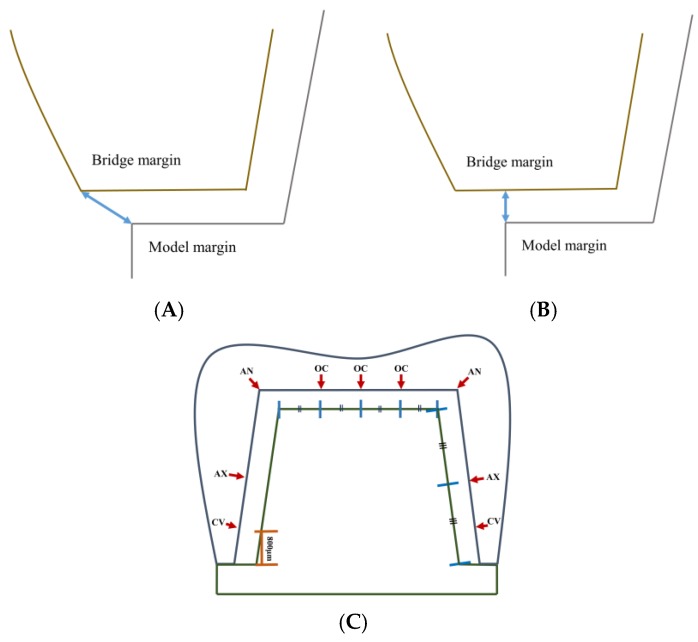
(**A**) The absolute marginal discrepancy (AMD) and (**B**) The marginal gap (MG) were measured for marginal fit. (**C**) The location of the internal gap length measurement points. Cervical area (CV) was measured at a height of 800 µm from the margin. Axial wall area (AX) was measured at mid-point of axial wall. Axio-occlusal angle area (AN) was measured at axio-occlusal angle. Occlusal area (OC) was measured at quadrants of occlusal plane.

Marginal fits were measured as absolute marginal discrepancy (AMD) and marginal gap (MG) according to the suggestion of Holmes et al. [28] (Figure 5A,B). Internal gap length was measured at the following sites [24] (Figure 5C): cervical area (CV) was measured at a height of 800 µm from the margin toward the occlusal plane; mid axial wall (AX) was measured at the midpoint of the axial wall; axio-occlusal angle (AN); occlusal area (OC) was measured at the center of the occlusal plane and at the midpoint between the center of occlusal plane and the axio-occlusal angel.

All measurements were taken at a magnification of 100×, and each measurement was repeated three times and the mean value was used.

### 2.6. Statistical Analysis

For each IGV, AMD, MG, CV, AX, AN, and OC, results were compared using two-way analysis of variance (ANOVA) with the confidence level of 95% (α = 0.05). The dependent variable was the gap and the two independent variables were the build orientation and layer thickness. Post-hoc tests were performed using Tukey’s HSD (honestly significant difference) test. When there was significant interaction between the two variables, statistical analysis was performed on one variable with other variable fixed. For each build orientation, the statistical significance between the layer thickness group was analyzed by independent t-test. For each layer thickness, One-way ANOVA was used to find out significance between the build orientations. Bonferroni correction was applied to correct errors that may occur in multiple comparisons. The 3D printing group with the smallest mean value was selected and compared with the milling group for each IGV, AMD, MG, CV, AX, AN, and OC separately. Independent t-test with the confidence level of 95% was used to analyze statistically (α = 0.05).

## 3. Results

### 3.1. Internal Gap Volume Analysis for 3D Printing

**Figure 6:**
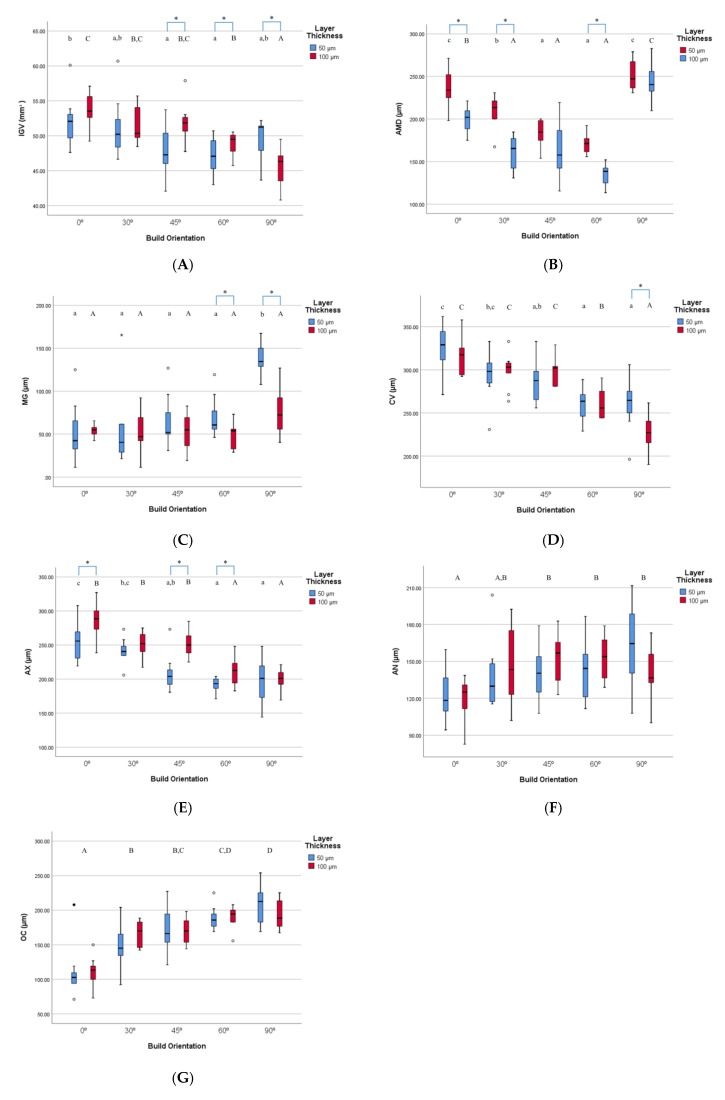
(**A**) The internal gap volume (IGV), (**B**) the absolute marginal discrepancy (AMD), (**C**) marginal gap (MG), (**D**) internal gap length at cervical area (CV), (**E**) internal gap length at axial wall area (AX), (**F**) internal gap length at axio-occlusal angle area (AN), (**G**) internal gap length at occlusal area (OC) for various 3D printing groups. Different lowercase letters in the 50 µm layer thickness group indicate statistically significant differences. Different uppercase letters in the 100 µm layer thickness group indicate statistically significant differences. * indicate statistical significance between different layer thickness group in same build orientation.

The internal gap volume (IGV) was the smallest in the 100 µm, 90° group to 45.5 ± 2.5 µm and the largest in the 100 µm, 0° group to 53.7 ± 2.6 µm. There was significant interaction between build orientation and layer thickness (*p* = 0.001). The significant difference according to the build orientation for each layer thickness and the significant difference according to the layer thickness for each build orientation was shown in Figure 6A. At the 50 µm layer thickness, the 60° build orientation was the smallest followed by the 45° build orientation and at the 100 µm layer thickness, 90° build orientation was significantly smaller than the other build orientations. At, 45° and 60° build orientations, the 50 µm layer thickness group was significantly smaller than the 100 µm layer thickness group, and at 90° build orientation, the 100 µm layer thickness group was significantly smaller than the 50 µm layer thickness group.

### 3.2. Marginal Fit and Internal Gap Length Analysis for 3D Printing

For each AMD, MG, CV, AX and OC, data was plotted as shown in Figure 6B–G. For AMD, the 100 µm, 60° group was the smallest value of 136.2 ± 11.8 µm. Two-way ANOVA showed that there was significant interaction effect between build orientation and layer thickness (*p* = 0.023). For the 50 µm layer thickness, the 45° and 60° groups were significantly smaller than the other groups. For the 100 µm layer thickness, the 30°, 45° and 60° groups were significantly smaller than the other groups. The 100 µm layer thickness group was significantly smaller than the 50 µm group at 0°, 30° and 60°.

For MG, the 100 µm, 60° group had the smallest value of 50.0 ± 14.7 µm. ANOVA results revealed that the interaction of the build orientation and the layer thickness was significant (*p* = 0.001). At the 50 µm layer thickness, the 90° group was significantly larger than the other groups. For 60° and 90°, the 100 µm layer thickness group was significantly smaller than the 50 µm layer thickness group.

For CV, the internal gap was the smallest in the 100 µm, 90° group to 227.6 ± 20.7 µm. There was an interaction between build orientation and layer thickness significantly (*p* = 0.026). At the 50 µm layer thickness, the 60° and 90° groups were significantly smaller than the other groups. At the 100 µm layer thickness, the 90° group was significantly smaller than the other groups. For 90°, the 100 µm layer thickness group was significantly smaller than the 50 µm layer thickness group.

The smallest internal gap for AX was 190.3 ± 11.3 µm in the 50 µm, 60° group. There was significant interaction effect between build orientation and layer thickness (*p =* 0.021). For both the 50 µm and the 100 µm layer thickness, the 60° and 90° groups were significantly smaller than the other build orientation groups. For 0°, 45° and 60°, the 50 µm layer thickness group was significantly smaller than the 100 µm layer thickness group.

For AN, the 100 µm, 0° group had the smallest value of 118.9 ± 17.5 µm. ANOVA showed that only build orientation (*p =* 0.001) significantly affects the internal gap at AN. The interaction of the build orientation and the layer thickness was not significant (*p =* 0.100). The 0° showed the smallest gap followed by 30° regardless of layer thickness.

For OC, the internal gap was the smallest in the 50 µm, 0° group to 111.2 ± 36.3 µm. The build orientation (*p =* 0.000) had a significant effect on the internal gap at OC, but the layer thickness (*p =* 0.775) had no significant effect. There was no significant interaction between build orientation and layer thickness (*p =* 0.341). The 0° showed the smallest gap followed by 30° regardless of layer thickness.

### 3.3. Comparison of 3D Printing and Milling

**Figure 7:**
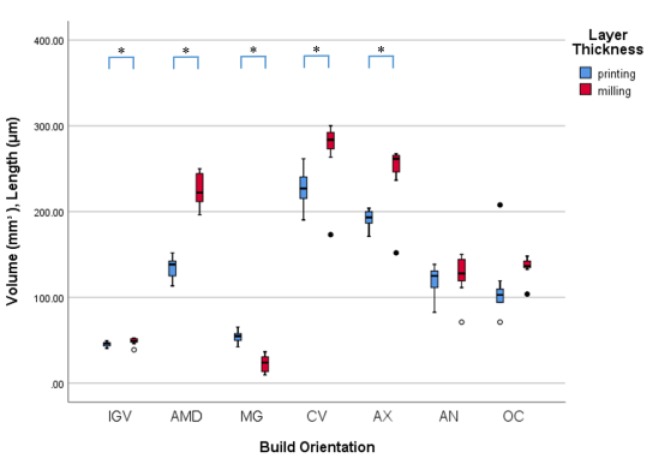
The internal gap volume (mm^3^), marginal fit and internal gap length (µm) of the selected 3D printing and the milling prostheses. * indicate statistical significance between the selected 3D printing group and the milling group.

For each IGV, AMD, MG, CV, AX, AN and OC, the value of the milling group and the smallest mean value in the 3D printing group were shown in Table 1. Data were plotted as shown in Figure 7.

For IGV, the mean value of the 100 µm, 90° group was the smallest for 3D printing and was selected to be compared with milling. The value of the milling group was 48.7 ± 4.0 mm^3^. The IGV value of 3D printing group was significantly smaller than those of milling group (*p* = 0.048).

The AMD value of 3D printing group was significantly smaller than those of milling group (*p* = 0.000). The MG value of the milling group was 23.1 ± 9.3 µm which was significantly smaller than those of the smallest 3D printing group (the 100 µm, 60° group) (*p* = 0.000).

For CV and AX, the value of the smallest 3D printing group was significantly smaller than the value of the milling group (*p* = 0.003 and *p* = 0.000, respectively).

## 4. Discussion

This study investigated the effect of build orientation and layer thickness on marginal fit and internal gap of 3-unit resin prosthesis fabricated using 3D printing. Also, the fit of prosthesis fabricated by 3D printing with optimal condition was compared with the fit of prosthesis manufactured by milling. The first null hypothesis that there was no difference in marginal fit and internal gap of 3D printed 3-unit resin prosthesis made with different build orientations and layer thickness was rejected. The second null hypothesis that there was no difference in marginal fit and internal gap between the prostheses made by 3D printing with optimal condition and milling was rejected. In this study, the 3-unit prostheses were fabricated to carry out the experiment assuming a more complex situation than single crown case.

There was no study to investigate the internal gap volume of the 3D printed prosthesis. In studies using lithium disilicate crown fabricated by CAD/CAM method, the internal gap volume was reported 12.6~18.2 mm^3^ by Yildirim et al. [29] and 25.3~40.7 mm^3^ by Kim et al. [30] The internal gap volume of present study was larger than that of the two studies because this study was investigated with bridge rather than a single crown. In the present study, the AMD value of the 3D printing with the smallest mean was 136.2 ± 11.8 µm and value of the milling was 225.1 ± 18.9 µm. The MG value of the 3D printing with the smallest mean was 50.0 ± 14.7 µm and the value of the milling was 23.1 ± 9.3 µm. According to the literature, the AMD value of single crown fabricated by CAD/CAM method was reported to be from 10 µm to 308 µm [31]. For the bridge framework, AMD value was reported to be from 9 µm to 206 µm [31]. The clinically acceptable limit for the marginal fit of conventional cast restoration to be up to 70 µm [3]. So, the MG values were well within the clinically acceptable limit for marginal fit of conventional cast restoration as well as fixed dental prosthesis. The internal gap length was analyzed in this experiment. For the bridge framework made by CAD/CAM method, the axial gap was reported between 9 µm and 140 µm, and occlusal gap between 68 µm and 280 µm [31]. Tuntiprawon and Wilson [32] reported that the cement thickness of more than 122 µm at the axial wall reduced the fracture resistance of crown. In the present study, large gap in cervical and axial area was probably due to the large cement space. There are few studies that have analyzed the fit of prosthesis depending on the build orientations. Tahayeri et al. [16] compared the accuracy of rectangular resin specimen manufactured by 3D printing according to the build orientation. In that study, the accuracy of the elements printed on the z-axis was high. For similar experiment, Unkovskiy et al. [17] reported that the z-axis printing parameter was significantly inaccurate which was in contradiction to Tahayeri et al. [16], and explained that the use of supporting structures might have influenced the results. Alharbi et al. [18] analyzed the accuracy of the 3D printed resin crown through three-dimensional superimposition technique, and concluded that 120° is the best build orientation condition considering the dimensional accuracy and time of finishing and polishing. (120° of that study is equal to 60° of present study). Osman et al. [8] conducted a similar experiment on crown made with DLP technique and obtained the best results at 135° (135° of that study is equal to 45° of present study).

In this experiment, the marginal fit and internal gap varied according to the build orientation. In addition, different results were obtained depending on the measured area of the internal gap. There are possible reasons for these results. First, depending on the build orientation, the area where the supporting structure connected to the prosthesis changes. Errors may occur due to an unsupported area, which changes depending on the build orientation [8]. Also, depending on the build orientation, the appearance of the layer output by the 3D printer changes. The DLP 3D printer polymerize one layer at a time. If the shape of the layer changes by build orientation, the form and degree of polymerization shrinkage would change. In addition, the accuracy of the 3D printer in the z-axis differs from the accuracy of the other axes [16,17]. For these various reasons, the marginal and internal gap showed different results depending on the build orientation in different area. In this study, the internal gap of 3D printed prosthesis, evaluated by IGV, was smaller at 45°, 60°, and 90° build orientations than those of the other build orientation. The marginal fit of 3D printed prosthesis, evaluated by AMD, was smaller at 45° and 60° build orientations than those of the other build orientation. The marginal fit of 3D printed prosthesis, evaluated by MG, was larger at 90° build orientations than those of the other build orientation. Taken together, a build orientation of 45° or 60° is recommended. The results also differ depending on the layer thickness. The internal gap volume was smaller at the 50 µm layer thickness than at the 100 µm layer thickness, but the marginal fit was greater at the 50 µm layer thickness than at the 100 µm layer thickness. This was probably due to the accumulation of errors due to more layers at the 50 µm layer thickness. In the present study, except for MG, the fit of milling prostheses showed a poor fit than the 3D printing with the smallest mean. These results are consistent with the results of Alharbi et al. [15]. The error due to the tolerance of the milling bur may reduce the accuracy of the milling group [4].

There are several ways to measure marginal gaps. Although the direct view method that uses the microscope to view the margin interface, is widely used but has several disadvantages [20]. It is impossible to measure the inner surface of the prosthesis and it is difficult to find the point to be measured [33]. In addition, errors due to the orientation of projection occur [34]. Cross-sectioning technique allows to see the marginal gap by sectioning the specimen in the desired orientation [20]. However, the specimen must be destroyed and measurements can be made only in a limited section [35]. Replica technique uses light-bodied silicone material instead of cement material. After removing the crown from die, it is filled with heavy-bodied silicone material and sectioned and observed with a microscope [25]. This method has drawbacks as well, it is difficult to figure out the crown margin and the silicone material may tear during the crown removal process [36]. In this study, marginal accuracy was measured using micro-CT. Images reconstructed in 3-dimensions can be used to measure the gap of invisible inner side of restoration without destroying the specimen [37]. Also, it is possible to measure at any angle or position through high quality images of 2 or 3-dimensions [29]. Images can be sectioned at very close proximity, allowing measurements at numerous positions [25]. If the difference in the radiopacity between two materials is too large, radiation artifacts may occur which make analysis difficult [29]. Conversely, if the difference in radiopacity is too small, it is difficult to find the interface, so that the interface of the object with the appropriate radiopacity difference is good for analysis [37]. The PMMA resin, 3D printing resin used in this experiment were suitable for micro-CT analysis because of the adequate radiopacity difference with empty space.

In this study, the model scanner was used for the 3D impression of the implant-supported 3-unit model. The model scanner is an extra-oral scanner. Currently, the extra-oral scanner includes touch probe scanner, laser scanner, white light scanner, and blue light scanner [38]. The scanner we used in our experiment was a white light scanner, which obtains three-dimensional information by projecting a specific pattern onto an object and determining the shape of the pattern [39].

In order to use 3D-printing manufacturing to fabricate the prosthesis in the dental filed, it is important to show a favorable IGV. The build orientation and the layer thickness were controllable parameters when 3D printing the prostheses. The purpose of this study was to find the 3D printing parameters which minimized the IGV of prostheses. So, the 3D printing parameters (the build orientation and the layer thickness) which showed the smallest value of the IGV we obtained in this experiment was chosen to be compared with the results of the milling method currently used in clinical fields.

We should consider the biomechanical behavior of the luting material because luting material seal the marginal gap. Sealing ability of luting material exposed to oral environment at marginal gap affects the extent of leakage [36]. Depending on the type of luting agent, physical properties and resistance to clinical stress are different and it affects the leakage at marginal gap [40]. The cement material may interfere with the complete seating of the prosthesis and can prevent sealing of the margin [40]. Stappert et al. [41] reported that the marginal gap before cementation in ceramic metal FDP was 53 μm and increased to 63 μm after cementation. Wolfart et al. [36] also reported that the marginal discrepancy of the crown increased from 96 μm to 130 μm after cementation. In this experiment, marginal gaps were measured under the prostheses without cementation. If the marginal gap was measured with cementation, the radiation artifacts could occur or the difference in radio-opacity might not be appropriate, so it would be difficult to measure the gaps. Further experiments are needed to determine the effect of luting agents on marginal gaps of 3D printed prosthesis.

In this study, preliminary experiments were conducted to find the appropriate cement space to passively fit the prosthesis to the model. The cement space of the prosthesis was evaluated by increasing from 0.04 mm by 0.02 mm, confirming the fit of the prosthesis on the model. The smallest value for all specimens passively seated on the model was 0.24 mm.

In this experiment, marginal fit was measured without cementation. More research is needed to investigate the effect of various luting agents on marginal fit. This study was performed on a 3-unit prosthesis. It is necessary to study how marginal fit changes in more complex prostheses in the future. In addition, there are various manufacturing methods besides the DLP method in the field of 3D printing, therefore, research regarding the IGV of the prosthesis fabricated by other manufacturing methods is also needed. Care should be taken in interpreting the results of this experiment. The results of this experiment may have different results depending on the equipment or materials used to produce the prosthesis. Experiments with a variety of equipment and materials are needed in the future. However, this study was one of the first researches to investigate the influences of the build orientation and the layer thickness on the fit of prosthesis, and the basic concept obtained from the study could be applied to help future studies.

## 5. Conclusions

Within the limitation of this study, the following conclusions were drawn. The fit of the 3D printed prosthesis varied depending on the build orientation and the layer thickness. Considering the marginal fit and the internal gap together, build orientation of 45° and 60° would be recommended. The marginal fit of 3D printed prosthesis with the 100 µm layer thickness was similar to that of the 50 µm layer thickness. When the prosthesis is manufactured by 3D printing with favorable parameters, it is possible to obtain a comparable fit to the prosthesis manufactured by the milling. The marginal fit of 3D printing and milling prostheses was within the clinically acceptable limit for marginal fit of conventional cast restoration.

## Figures and Tables

**Table 1:** The internal gap volume (mm^3^), marginal fit and internal gap length (µm) of the selected 3D printing and milling prosthesis. * indicate statistical significance between 3D printing group and milling group.

Area	Selected 3D Printing Group	3D Printing	Milling	*p*-Value
IGV	100 µm, 90°	45.5 ± 2.5	48.7 ± 4.0	* *p* = 0.048
AMD	100 µm, 60°	136.2 ± 11.8	225.1 ± 18.9	* *p* = 0.000
MG	100 µm, 0°	50.0 ± 14.7	23.1 ± 9.3	* *p* = 0.000
CV	100 µm, 90°	227.6 ± 20.7	273.4 ± 37.0	* *p* = 0.003
AX	50 µm, 60°	190.3 ± 11.3	247.2 ± 35.0	* *p* = 0.000
AN	100 µm, 0°	118.9 ± 17.5	125.4 ± 22.7	*p* = 0.480
OC	50 µm, 0°	111.2 ± 36.3	135.4 ± 12.2	*p* = 0.06
